# MSC-derived extracellular vesicles accelerate wound healing in senescent fibroblast cultures

**DOI:** 10.1007/s10522-026-10468-3

**Published:** 2026-07-02

**Authors:** Ekaterina Rudnitsky, Naomy Vineshtock, Natali Yakubov, Alex Braiman, Yael Segev, Marina Wolfson, Khachik K. Muradian, Vera Gorbunova, Gadi Turgeman, Michaela Ben Shahar, Vadim E. Fraifeld

**Affiliations:** 1 The Shraga Segal Department of Microbiology, Immunology and Genetics, Faculty of Health Sciences, Ben-Gurion University of the Negev, 8410501 Beer-Sheva, Israel; 2 Department of Biology of Aging and Experimental Life Span Extension, State Institute of Gerontology of National Academy of Medical Sciences of Ukraine, Kiev, 4114 Ukraine; 3 Departments of Biology and Medicine, Rochester Aging Research Center, University of Rochester, Rochester, NY 14627 USA; 4 Department of Molecular Biology, Faculty of Natural Sciences and The Adelson School of Medicine, Ariel University, 40700 Ariel, Israel

**Keywords:** Mesenchymal stem cells (MSCs), Extracellular vesicles (EVs), Wound healing, Fibroblasts, Cellular senescence

## Abstract

Wound healing (WH) is a multi-component and highly orchestrated process, in which fibroblasts play a pivotal role. Wound healing is commonly attenuated during aging and the course of cellular senescence (CS). A growing body of evidence demonstrates that mesenchymal stem cell (MSC)-derived extracellular vesicles (EVs) can mitigate the aging and CS phenotype. In this study, we evaluated the effects of EVs derived from naïve MSCs (nMSCs) or anti-inflammatory polarized MSCs (pMSCs) on the rate of in vitro wound healing in primary cultures of human pulmonary fibroblasts (HPF) at various stages of cellular senescence. The major finding of this study is that MSC-derived EVs increase the rate of in vitro WH in senescent but not young HPF cultures. Yet, the pMSC-derived EVs had a stronger impact on WH acceleration in senescent cultures as compared to the nMSC-derived EVs, and this impact was apparently attributed to cell migration.

**Supplementary Information:**

The online version contains supplementary material available at https://doi.org/10.1007/s10522-026-10468-3.

## Introduction

Wound healing (WH) is a fundamental biological process needed for recovery of integrity and functionality of a tissue after injury. WH is a highly-orchestrated process which includes migration of cells from edges of the wound (so-called ‘leadindsg edge’) to each other followed by proliferation of the cells behind the leading edge (so-called ‘proliferative hub’) (Zanca et al. 2022). Not surprisingly, because of its complexity, WH may be affected by various conditions, including cellular senescence (CS) and aging. It was observed in various animal models that the rate of wound closure decreases with the advanced age (for review see Yanai et al. 2011; Kremer and Burkemper 2024). Deviations from regular WH could lead to diverse age-related pathological conditions, while recovery of WH in aged tissues to the youthful levels is of great importance for healthy longevity (Yanai et al. 2015, 2016). Thus, the controlled modulation of WH represents a major biomedical challenge (Bogadi et al. 2025).

In biogerontology, mesenchymal stem cells (MSCs) have attracted much attention because of their longevity-promoting properties (Rudnitsky et al. 2025), availability, low immunogenicity, etc. (Chen et al. 2023). MSCs exert their effects primarily through a paracrine mechanism (Gnecchi et al. 2016; Gobshtis et al. 2017, 2021; Tfilin et al. 2023), thus suggesting the release of bioactive compounds as individual molecules or encapsulated in extracellular vesicles (EVs). Various modifications of either MSCs or EVs may strengthen anti-aging effects of EVs (Abuzan et al. 2025). One of such modifications is polarisation of MSC cultures towards anti-inflammatory phenotype. Previously, we have shown that MSC polarized to an anti-inflammatory MSC2 phenotype can increase hippocampal neurogenesis and improve cognitive function in aged mice (Tfilin et al. 2023). The promoting effects of MSC-derived EVs on skin WH have been recently demonstrated (Tutuianu et al. 2021; for review see Porwal et al. 2025). Despite a crucial impact of CS on the rate of WH, this issue has not thus far been investigated with regard to the impact of MSC-derived EVs.

In vitro WH is a suitable and widely used model for evaluation of basic processes and mechanisms of action of various agents on the wound closure (Planz et al. 2018). Here, we used primary cultures of human pulmonary fibroblasts (HPFs) for modelling WH and evaluating the efficacy of EVs derived from naïve MSCs (nMSCs) or anti-inflammatory polarized MSCs (pMSCs), on WH rate at various stages of cellular senescence.

## Materials and methods

### Cell culturing and counting

Primary cultures of HPFs (obtained from ScienCell Cat.#3300; Carlsbad, CA, USA) were grown under standard conditions (37 °C, 5% CO^2^) in Dulbecco’s modified Eagles medium (DMEM) (Cat.#01-055-1A), supplemented with 10% fetal bovine serum (Cat.#04-121-1A), 1% L-glutamine (Cat.#03-020-1B), and 1% penicillin/streptomycin (Cat.#03-031-5B). All products for cell cultures were obtained from Biological Industries, Beit Haemek, Israel. The cultures were inspected daily under an inverted phase-contrast microscope (Primo Vert, Zeiss, Oberkochen, Germany), and cells were passaged 1:2 upon reaching 75%–80% confluence. The number and concentration of viable cells were calculated using the Trypan blue (Cat.# 15,250–061; Life Technologies, Grand Island, NY, USA) exclusion assay.

### The model of replicative CS

CS was achieved by serial passaging. The cultures were defined as pre-senescent, senescent, or deep senescent based on (i) a gradual slowing (up to ceasing) of cell growth, (ii) typical CS morphology, and (iii) expression of senescence-associated β-galactosidase (SA-β-gal), commonly accepted marker of senescent cells. The SA-β-gal assay was conducted on a six-well plate (50,000 cells per well), according to the manufacturer’s protocol (Senescence Cells Histochemical Staining Kit Cat.#CS0030; Sigma-Aldrich, St. Louis, MO, USA). The cells were incubated with the Staining Mixture overnight at 37 °C. SA-β-gal staining was visualized using an inverted phase-contrast microscope.

### MSC-derived extracellular vesicles

**Fig. 1:**
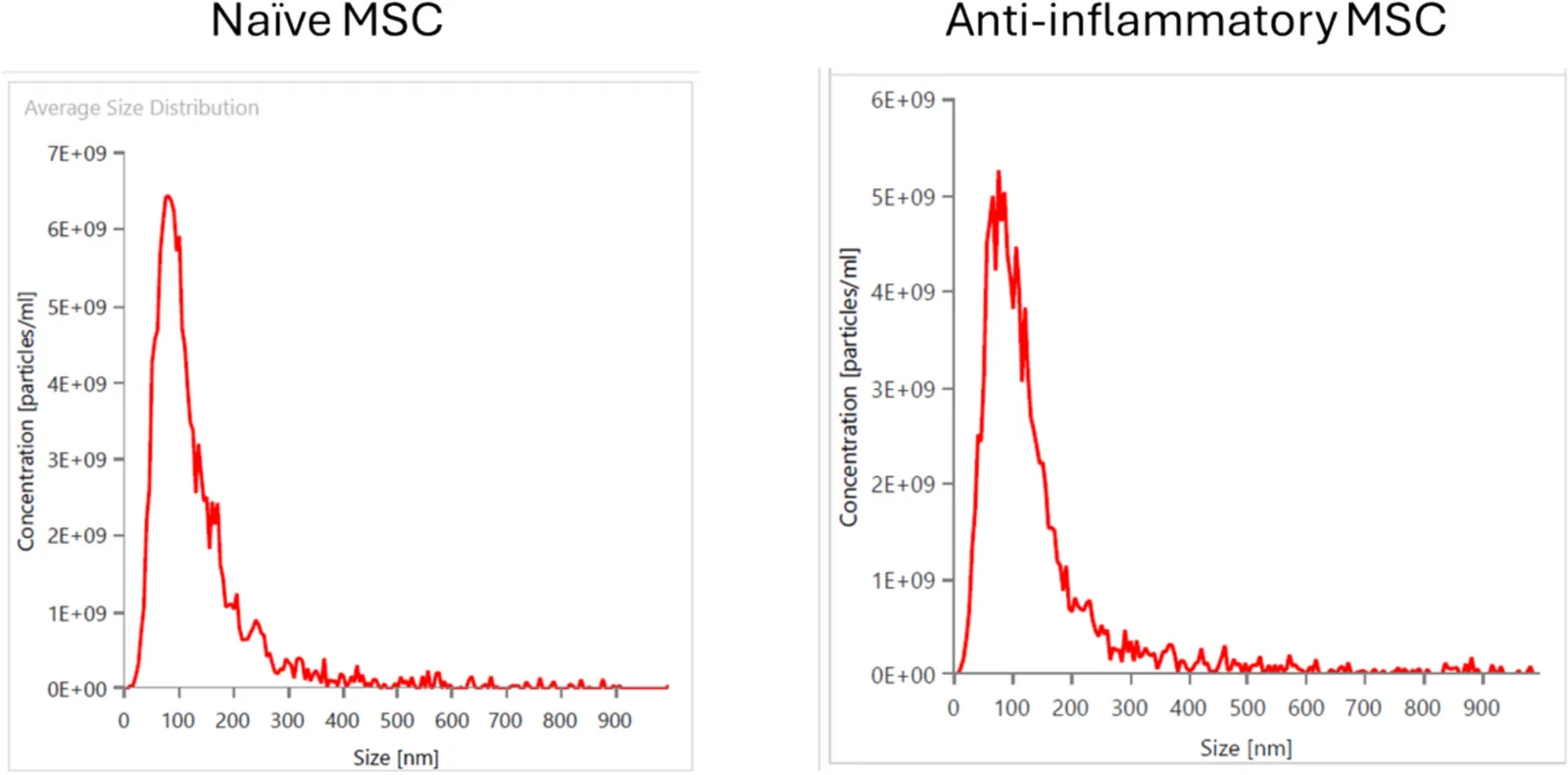
Size distribution of MSC-isolated EVs

Human bone marrow-derived MSCs were purchased from ATCC (PCS-500-012) and polarized to MSC2 phenotype (pMSC) by adding PACAP-27 (Cat.#4,031,084; BACHEM AG, Bubendorf, Switzerland) to the culture media for 5 days, at a final concentration of 20 nM, as we previously described (Tfilin et al. 2023). Conditioned medium was collected following 48 h of incubation, with DMEM, supplemented with 1% L-glutamine and 1% penicillin/streptomycin and 10% EV-depleted FBS (Cat.#S140M-050; Biowest, Nuaillé, France). Extracellular vesicles were isolated from conditioned media of nMSC or pMSC cultures at passages 13–15. Conditioned media was centrifuged at 300 g for 5 min to remove floating and dead cells, followed by additional centrifugation at 3000 g for 10 min to remove debris. EVs were isolated from the supernatant by tangential flow filtration using TFF-EV filter cartridge (HansaBioMed, Tallin, Estonia) according to manufacturer’s instructions. EVs were characterized and quantified by Nanoparticle Tracking Analysis using NanoSight NS300 (Malvern Panalytical, Malvern, UK), with EV size ranging mainly from 48 to 165 nm (Fig. 1).

### EV administration

HPF cultures of various states (young: population doubling time [PDT] = 1 day, passage 15 [P.15]; pre-senescent: PDT = 1 week, P.40; senescent: PDT = 2–3 weeks, P.50; deep senescent: cells ceased to divide, P.60) were seeded in 6-well plate (100,000 cells per well) and supplemented by EVs derived from nMSCs or pMSCs at the concentration of 10^6^ EVs per 1 mL medium for 72 h prior WH assay.

### Wound healing assay

To analyse the rate of WH, 5 × 10^4^ fibroblasts were seeded into two silicon chambers divided by a separator of the standard width (Culture-insert Cat.#80,209; Ibidi GmbH, Gräfelfing, Germany). After appropriate cell attachment (24 h) the chambers were removed, and the rate of wound closure was visualized and evaluated daily using an inverted phase-contrast microscope.

### Fluorescence activated cell sorting analysis

Fluorescence activated cell sorting (FACS) was used for quantification of cells in various phases of cell cycle as well as for quantification of dying cells in HPF cultures. Cells and incubation medium were collected. For cell cycle assay, the cells were fixed by 70% ethanol. After two washings with PBS cells were incubated with RNAse A (Cat.#AC118; Omega Bio-Tek, Norcross, GA, USA) at 37 °C for 20 min. Then propidium iodide (PI) solution–0.1 mg/ml PI (Cat.#P4170) in 0.6% Triton-X100 (Cat.#03–0013584) both from Sigma-Aldrich, St. Louis, MO, USA, in PBS–was added, and cells were incubated at 37 °C for 20 min. After washing with PBS, a FACS buffer was added to the cells. For the analysis of cell death, cells were washed twice with PBS. Then the cells were incubated with FITC Annexin V antibodies and PI (eBioscience Annexin V Apoptosis Detection Kit Cat.#88-8005-72, ThermoFisher Scientific, Waltham, MA, USA) at RT for 15 min. The FACS analysis was performed using the CytoFLEX system (Beckman Coulter Life Sciences, Indianapolis, IN, USA), and the results were analysed by FlowJo software V10. For cell cycle assay, the cells were gated using the strategy illustrated in Fig. S1A. The intensity of PI staining was measured at Ex. 488 nm, Em. 575–610 nm. The gates for cell cycle phases were selected based on cellular DNA content. For cell death assay, the cells were gated using the strategy illustrated in Fig. S1B. The intensity of FITC Annexin V antibodies (Ex. 488 nm, Em. 530 nm) and PI (Ex. 488 nm, Em. 575–610 nm) was measured. Distinguish was made between viable cells (Annexin V − /PI −), apoptotic cells (Annexin V +), and necrotic cells (Annexin V − /PI +) as described elsewhere (Wlodkowic et al. 2009).

### mRNA isolation and quantitative real-time polymerase chain reaction (qRT-PCR)

RNA was extracted from cell pellets by the Blood/Cell Total RNA Mini Kit (Cat.#RB300; Geneaid, New Taipei City, Taiwan), and 1 μg of total RNA was transcribed into cDNA in 20 μl reactions using the qScript cDNA Synthesis Kit (Cat.#95,047; Agantek, Yakum, Israel). qRT-PCR was performed using the SYBR Green Gene expression assays qPCRBIO SyGreen Blue Mix on the MIC 2 channel qRT-PCR system (Bio Molecular Systems; Upper Coomera, Australia), according to the manufacturer’s instructions. In order to detect the expression of selected genes, multiple primer pairs were designed by the Primer-BLAST online tool (Ye et al. 2012) and analysed while the *GAPDH* gene was used as a normalization control (Table 1).

**Table 1:** Primers used for RT-qPCR analysis

Gene	Primer sequence (5’-3’)
*BCL2*	Forward: AGGCATCCCAGCCTCCGTTA Reverse: TGTGTGTGGAGAGCGTCAACC
*CCND* (Cyclin D)	Forward: CGCAATGACCCCGCACGATTT Reverse: TGCGCGTGTTTGCGGATGAT
*CDKN2A* (p16)	Forward: CCACCCCGCTTTCGTAGTT Reverse: AGTGAAAAAGGCACAAGCGGT
*C-MET*	Forward: AGCCAACCGAGAGACAAGC Reverse: ACCTGTTATTGTGCTCCCACC
*HGF*	Forward: TTCTTTCACCCAGGCATCTCC Reverse: TCTTTTCCTTTGTCCCTCTGCAT
*VIM* (Vimentin)	Forward: AGGCGAGGAGAGCAGGATTT Reverse: TCGTGATGCTGAGAAGTTTCGT
*GAPDH*	Forward: ACATCGCTCAGACACCATG Reverse: TGTAGTTGAGGTCAATGAAGGG

### Statistical evaluation

The effects of CS stage and MSC-derived EVs were evaluated using two-way ANOVA. The difference between experimental and control groups was evaluated using a two-tailed Student’s t-test. For WH, the area under the curve (AUC) was estimated. P-values < 0.05 were considered statistically significant.

## Results

### Rate of *in vitro* wound healing during the course of cellular senescence

**Fig. 2:**
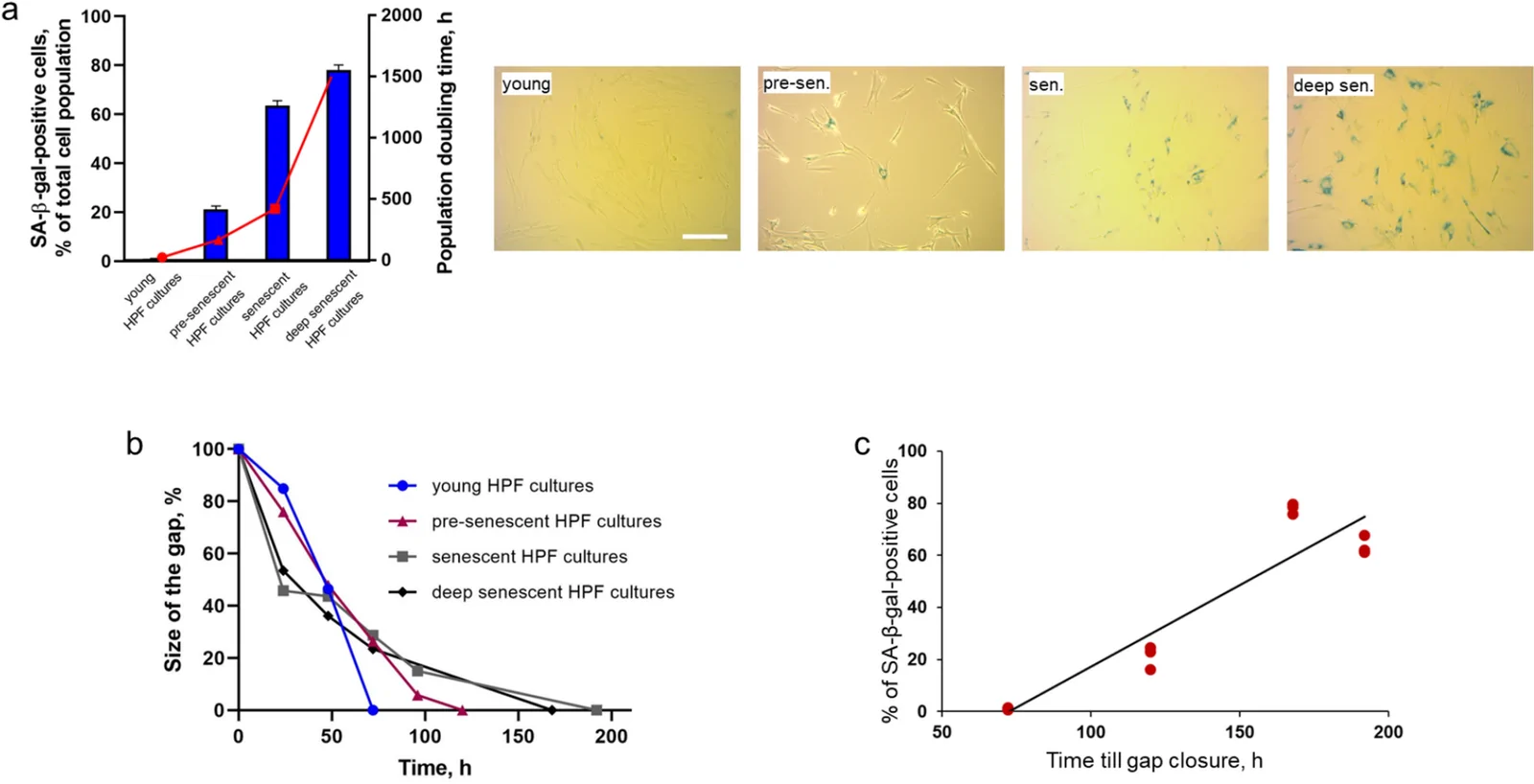
The rate of wound closure during the course of cellular senescence in HPF cultures. **a**–the graph showing that the percentage of SA-β-gal-positive senescent cells (columns) increased together with PDT (line) through passaging of fibroblasts, and representative images of SA-β-gal staining; the data is presented as mean ± SEM (n = 6 independent replicates; differences between passages are highly significant, p < 0.001), the scale bar is 200 μm; **b**–the rate of wound closure in HPF cultures of various passages, the data is presented as mean (n ≥ 3 independent replicates); **c**–positive correlation between time needed for wound closure and the amount of senescent cells in HPF cultures (R^2^ = 0.857; R = 0.925, p < 0.0001)

Two phenotypic markers of CS—population doubling time (PDT) and accumulation of SA-β-gal-positive cells—significantly increased with the number of passages in primary cultures of HPFs. Highly proliferative young HPF cultures (PDT 1 day, passage 15 [P.15]) contained negligible amounts of SA-β-gal-positive senescent cells (Fig. 2a). The percentage of SA-β-gal-positive cells dramatically increased in pre-senescent (PDT 1 week, P.40), further elevated in senescent (PDT 2–3 weeks, P.50), and reached its maximum in deep senescent (cells ceased to divide for at least 2 months, P.60) HPF cultures.

**Fig. 3:**
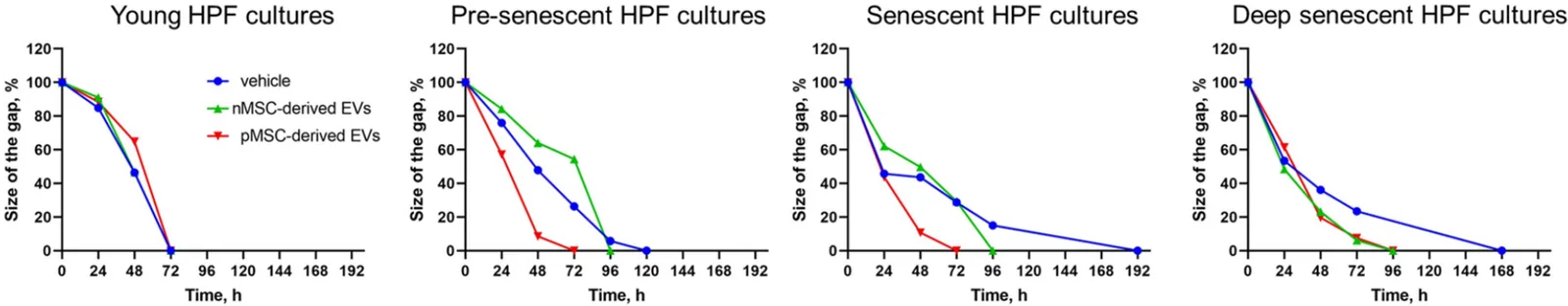
The rate of wound healing in HPF cultures of various passages with or without EV supplementation. The data is presented as means of at least 3 independent replicates

As seen in Fig. 2b, the higher the CS stage, the slower the rate of in vitro WH (one-way ANOVA for AUC: p < 0.01). Accordingly, the time needed for wound closure positively correlated with the amount of senescent cells in HPF cultures (R^2^ = 0.857; R = 0.925, p < 0.0001; Fig. 2c). Notably, the shape of the curve, fitting the wound closure dynamics, changed in the course of CS (Fig. 2c; see also Fig. 3 vehicle). In young HPF cultures, a squared function is the most fitting shape (y = –0.01631x^2^ + 0.261x + 101.67; R^2^ = 0.991), whereas in senescent HPF cultures, the wound closure process is better described by exponential function (y = 98.33 × e^–0.0218x^; R^2^ = 0.993). This could be attributed to different contributions of cell proliferation and cell migration to WH at various stages of CS.

### nMSC/pMSC-derived EVs accelerate wound healing in senescent but not in young HPF cultures

Supplementation of HPF incubation medium with either nMSC- or pMSC-derived EVs 72 h prior wounding, led to a significant acceleration of cell gap closure in senescent but not young HPF cultures (Fig. 3). Of note, in this regard, pMSC-derived EVs were more efficient than EVs derived from nMSCs (t-test for AUC: p < 0.0001 in pre-senescent and senescent HPFs).

### The effects of nMSC/pMSC-derived EVs on cellular senescence

**Fig. 4:**
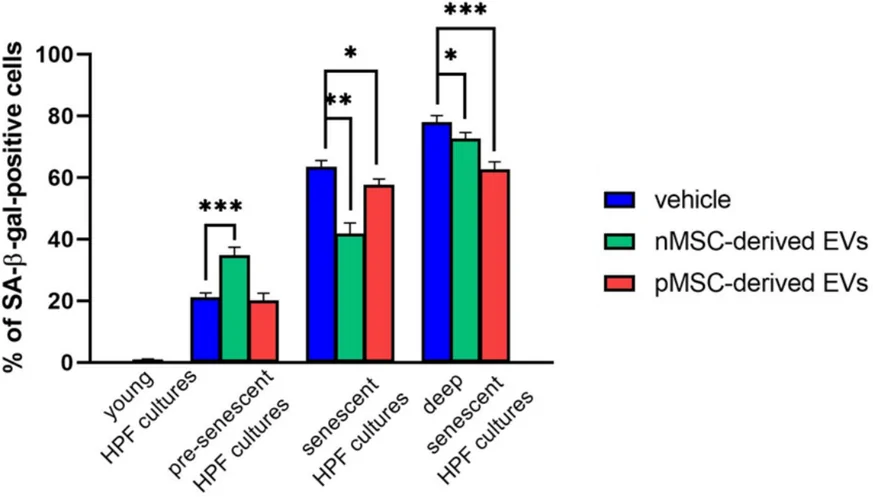
EVs derived from nMSCs/pMSCs decreased the percentage of senescent cells in senescent and deep senescent HPF cultures. The data is presented as mean ± SEM (n ≥ 3 independent replicates). * p < 0.05; ** p < 0.01; *** p < 0.001

The observed effects of EVs on the rate of WH in senescent HPF cultures may be mediated by their potential anti-senescent action. Indeed, both nMSC- and pMSC-derived EVs decreased the number of senescent cells in senescent and deep senescent HPF cultures (Fig. 4). Surprisingly, EVs derived from pMSCs did not affect the number of senescent cells in pre-senescent cultures, whereas nMSC-derived EVs even increased this parameter.

### The effects of nMSC/pMSC-derived EVs on cell cycle

**Fig. 5:**
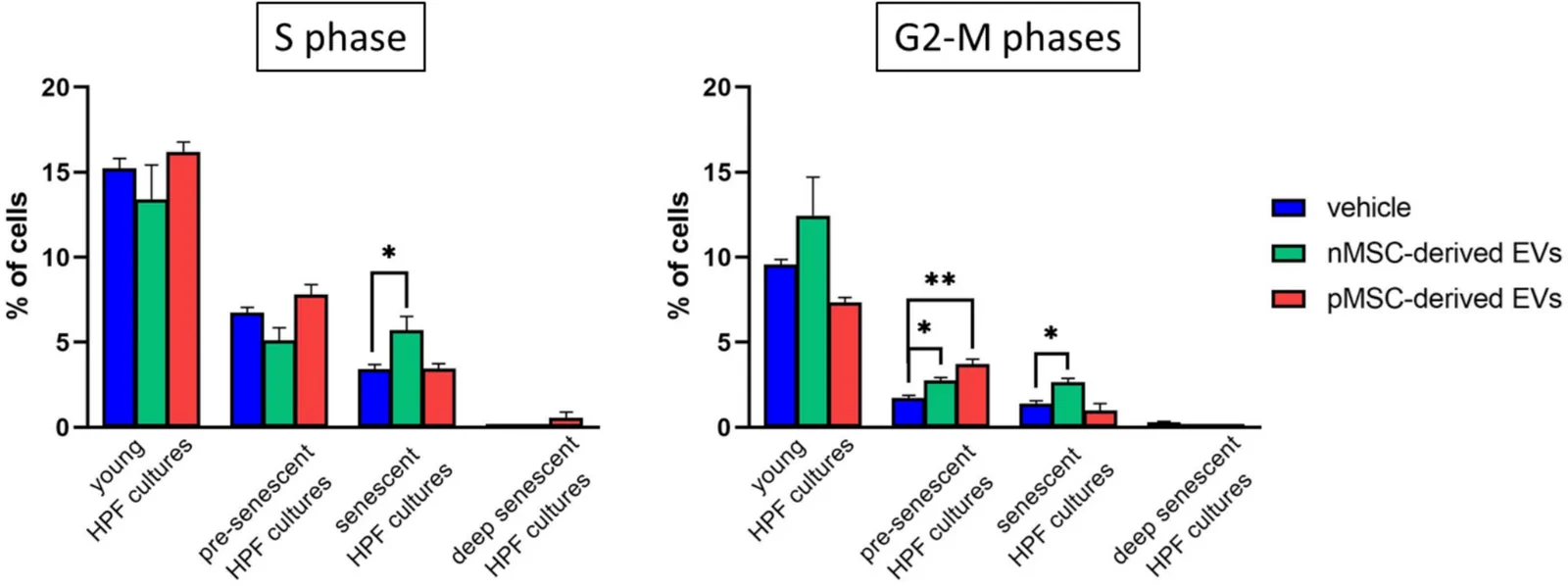
The percentage of cells at S and G2-M phases of cell cycle in HPF cultures of various passages with or without EV supplementation. The data is presented as mean ± SEM (n ≥ 3 independent replicates). * p < 0.05; ** p < 0.01

As expected, the percentage of fibroblasts in S and G2-M phases of cell cycle significantly decreased with the number of passages (one-way ANOVA: p < 0.0001; Fig. 5). Supplementation of the incubation medium with either nMSC- or pMSC-derived EVs significantly modified these parameters (Fig. 5). In pre-senescent HPF cultures, administration of both nMSC- and pMSC-derived EVs led to an increase in the percentage of cells in G2-M phases. In senescent HPF cultures, nMSC-derived EVs increased the percentage of cells in S and G2-M phases (Fig. 4), indicating activation of cell proliferation. HPF cultures in deep senescence did not respond to either nMSC- or pMSC-derived EVs in the context of cell cycle.

### The effects of nMSC/pMSC-derived EVs on cell death

**Fig. 6:**
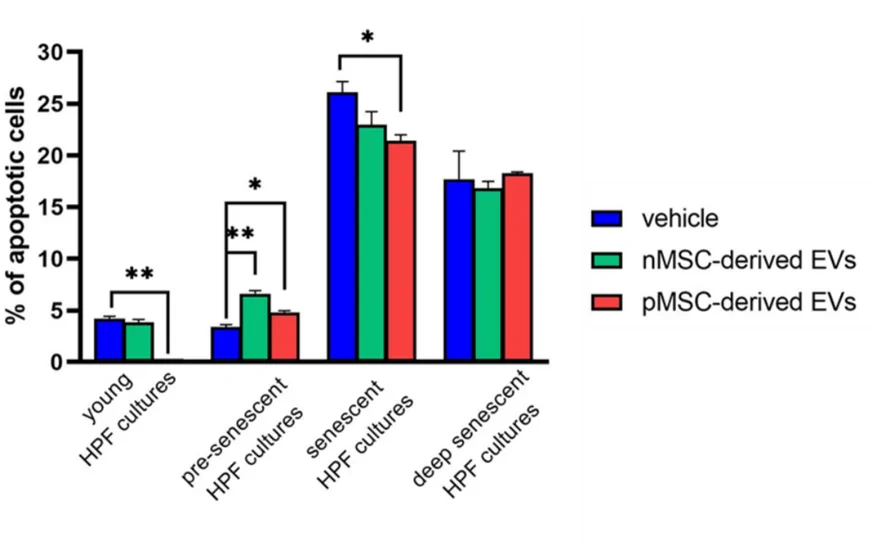
The percentage of cells dying through apoptosis in HPF cultures of various passages with or without EV supplementation. The data is presented as mean ± SEM (n ≥ 3 independent replicates). * p < 0.05; ** p < 0.01

Next, we analysed the amount of dying cells. It should be noted that only a negligible amount of cells died through necrosis in all groups (the data is not shown), so we focused on apoptotic cell death. As seen in Fig. 6, in young HPF cultures pMSC-derived EVs significantly suppressed apoptosis. Unexpectedly, both nMSC- and pMSC-derived EVs increased apoptosis in pre-senescent HPF cultures. In senescent HPF cultures, pMSC-derived EVs exerted an anti-apoptotic effect (Fig. 6).

### Expression of selected genes in HPF cultures after EV administration

**Fig. 7:**
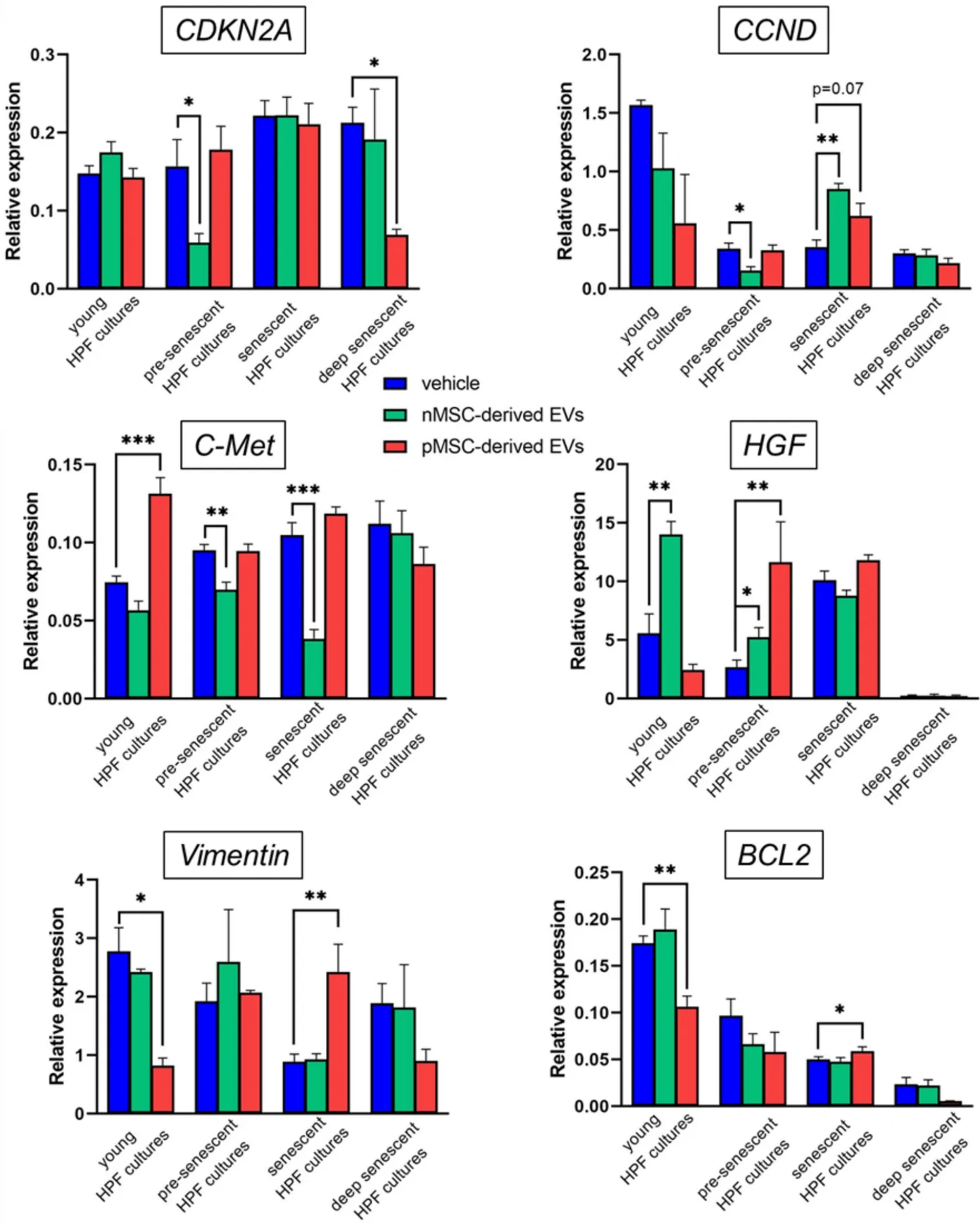
Expression of genes in HPF cultures of various passages with or without EV supplementation. The data is presented as mean ± SEM (n ≥ 3 independent replicates). *−p< 0.05; ** p < 0.01; *** p < 0.001

First, we analysed the expression of *CDKN2A* and *CCND* genes, as encoded proteins (p16 and Cyclin D, respectively) regulate G1/S checkpoint transition during cell cycle. As expected, the expression of *CDKN2A* increased (one-way ANOVA: p < 0.03) and the expression of *CCND* decreased (one-way ANOVA: p < 0.006) with the number of passages (Fig. 7). Interestingly, in pre-senescent HPF cultures nMSC-derived EVs decreased the expression of both genes (Fig. 7). In senescent HPF cultures, both nMSC- and pMSC-derived EVs increased the expression of *CCND* gene, and in deep senescent HPF cultures pMSC-derived EVs decreased the expression of *CDKN2A* gene. Taken together, these results testify the anti-CS action of MSC-derived EVs, which was more pronounced in the case of EVs derived from pMSCs.

Then we analysed the expression of *HGF* and its receptor *C-Met* because of their role in WH and CS (Li et al. 2013; Boichuck et al. 2019). The results confirmed that the expression of *C-Met* increased with passaging (one-way ANOVA: p < 0.02). Expression of *HGF* was also significantly influenced by stage of CS (one-way ANOVA: p < 0.0001): it significantly increased in senescent HPF cultures and then drastically dropped in non-dividing deep senescent ones (Fig. 7). EVs derived from nMSCs activated HGF signalling pathway (that is, increased *HGF* expression) in young HPF cultures, compensated its activity in pre-senescent HPF cultures (increased *HGF* expression and decreased *C-Met* expression), and down-regulated the pathway in senescent cultures (that is, decreased *C-Met* expression). EVs derived from pMSCs activated HGF signaling pathway in young and pre-senescent HPF cultures by an increase in *C-Met* or *HGF* expression. Both nMSC- and pMSC-derived EVs did not affect the expression of *HGF* and *C-Met* in deep senescent cultures (Fig. 7).

Vimentin (*VIM*) expression also significantly decreased with passaging (one-way ANOVA: p < 0.005). Two-way ANOVA revealed CS-dependent effects of EVs derived from pMSCs on *VIM* expression (p < 0.0001; Fig. 7). This was not observed in the case of nMSC-derived EVs.

Expression of anti-apoptotic *BCL2* gene significantly decreased with passaging (one-way ANOVA: p < 0.0001; Fig. 7) which is in line with results on the number of dying cells (Fig. 6). EVs derived from pMSCs decreased *BCL2* expression in young HPF cultures and increased it in senescent ones (Fig. 7). EVs derived from nMSCs did not significantly affect *BCL2* expression.

## Discussion

The gradual slowing of the WH rate is indicative of the aging process and was reported for various mammalian species, humans included (Yanai et al. 2016). Moreover, the ability to preserve the rate of WH up to an old age appears to be associated with a longevity phenotype. A positive relationship between the rate of WH and the longevity in various murine strains was true only for advanced but not the young ages (Yanai et al. 2011, 2015, 2016). Thus, any promotion of WH in the advanced age could be considered one of the anti-aging strategies.

Accumulation of senescent cells is one of the hallmarks of aging (López-Otín et al. 2013, 2023). Although there is evidence indicating the necessity of senescent cells in WH (Demaria et al. 2014; Avelar et al. 2020; Walters et al. 2023), its rate dramatically decreases in the course of CS (Yanai et al. 2015; Lavarti et al. 2025). In vitro model of WH is widely used for investigating the mechanisms of wound closure (Ud-Din and Bayat 2017; Planz et al. 2018). In vitro models are also well-recognized tools for investigating CS (Rattan 2012). Nevertheless, any in vitro model will a priori be a substantial simplification. It is also true regarding fibroblast cultures, despite a pivotal role of fibroblasts in WH. Realizing all this, we used primary cultures of human pulmonary fibroblasts as an in vitro model of WH to get an insight into the superimposed effects of MSC-derived EVs and CS. Putting in other words, we tried to answer the question, whether the effect of nMSC/pMSC-derived EVs on the rate of WH depends on the CS stage of HPF cultures.

The major finding of this study is that MSC-derived EVs increase the rate of in vitro WH in senescent but not young HPF cultures (Fig. 3). This effect may be mediated by their anti-CS action (Fig. 4). Interestingly, this coincides well with our previous in vivo observation in murine models (Yanai et al. 2011, 2015, 2016). The rate of WH is determined by the two major processes, cell proliferation and cell migration, which could have a different “weight” at various stages of CS and could be described by different mathematical functions (for review see: Arciero and Swigon 2021). In young HPF cultures, both cell migration and cell proliferation contribute to cell gap closure. In contrast, in senescent cultures, cell proliferation has a low (if any) impact, and wound closure occurs predominantly by cell migration that apparently is reflected by different types of functions: a squared function in the case of young cultures and an exponential function in senescent ones (Figs. 2b and 3). Interestingly, in nMSC-derived EV-treated pre-senescent and senescent (but not deep senescent) HPF cultures, the wound closure curve exhibits an intermediate shape between the young and senescent cultures (Fig. 3), indicating a possible increase in the contribution of cell proliferation after the treatment with nMSC-derived EVs. This is supported by the data on the nMSC-derived EV-induced changes in the cell cycle. Indeed, the fraction of cells in S and G2-M phases of cell cycle as well as Cyclin D (*CCND*) expression significantly increased in senescent HPF cultures (Figs. 5 and 7). Intriguing results were obtained on effects of nMSC-derived EVs in pre-senescent HPF cultures: an increased number of senescent cells was accompanied by increased apoptosis, decreased expression of both *CDKN2A* and *CCND* and an increase in number of cells in G2-M phases of cell cycle. The possible explanation of this apparent inconsistency may be that in pre-senescent cultures nMSC-derived EVs affected G2-M checkpoint rather than G1-S. Thus, the cells successfully pass G1-S which is evident by preserved Cyclin D/p16^INK4A^ ratio, but then could not overcome G2-M checkpoint, accumulate at G2-M phases, and finally undergo senescence or apoptosis. P16^INK4A^-independent senescence was already shown for alterations of S phase progression (Prieur et al. 2011).

As compared to nMSC-derived EVs, the pMSC-derived EVs had a stronger impact on WH acceleration in senescent cultures, and, according to the shape of the curve fitting the cell gap closure, this impact was mostly attributed to cell migration (Fig. 3). It is worth emphasizing that in pre-senescent HPF cultures pMSC-derived EVs exerted their effects more through activation of HGF / C-met pathway (Fig. 7) which is known to be involved in migration and proliferation (Wang et al. 2023), whereas in senescent HPF cultures pMSC-derived EVs recruit Vimentin expression (Fig. 7). Of note, the rate of wound closure in senescent HPF cultures supplemented with pMSC-derived EVs was similar to that of young HPF cultures, thus exerting a clear rejuvenative effect.

To clarify if MSC-derived EVs exert senolytic or senomorphic effects on senescing HPF cultures, we analysed the amount of dying cells. An increase in the number of apoptotic cells was shown as functions of passage number in fibroblast cultures (Mammone et al. 2006), and here we confirmed this observation (Fig. 6). We did not observe any correlation between the amounts of senescent cells and dying cells in EV-treated HPF cultures of various stages of CS. These results led us to conclusion that nMSC/pMSC-derived EVs exerted rather senomorphic than senolytic effect on senescent fibroblasts.

In summary, both nMSC- and pMSC-derived EVs display a significant impact on WH in senescing HPF cultures. This impact differs in HPF cultures treated with nMSC- or pMSC-derived EVs, which presumably is attributed to their different effects on the major WH components (cell migration or proliferation). This in turn could, to a great extent, be induced by the protein content of EVs and differential expression of key WH-related genes by fibroblasts. Further in vivo investigation is warranted to evaluate whether MSC-derived EVs are able to improve WH in old age.

## Supplementary Information

Below is the link to the electronic supplementary material.

Supplementary file1 (DOCX 1813 KB)

## Data Availability

No datasets were generated or analysed during the current study.

## Abbreviations

AUC: Area under the curve
CS: Cellular senescence
EVs: Extracellular vesicles
FACS: Fluorescence activated cell sorting
HPFs: Human pulmonary fibroblasts
nMSCs: Naïve Mesenchymal stem cells
P.: Passage
PDT: Population doubling time
PI: Propidium Iodide
pMSC: Polarized Mesenchymal stem cells
SA-β-gal: Senescence-associated β-galactosidase
WH: Wound healing
