# Supplementary material for: MSC-derived extracellular vesicles accelerate wound healing in senescent fibroblast cultures
Source: Biogerontology. 2026 Jul 2;27(4):121. doi: 10.1007/s10522-026-10468-3 (PMC13328317; doi:10.1007/s10522-026-10468-3)
Supplement: Supplementary file 1 — Supplementary file1 (DOCX 1813 KB) [file 10522_2026_10468_MOESM1_ESM.docx]

**MSC-derived extracellular vesicles accelerate wound healing in senescent fibroblast cultures**

Ekaterina Rudnitsky^a,#,*^, Naomy Vineshtock^a,#^, Natali Yakubov^a^, Alex Braiman^a^, Yael Segev^a^, Marina Wolfson^a^, Khachik K. Muradian^b^, Vera Gorbunova^c^, Gadi Turgeman^d^, Michaela Ben Shahar^d^, Vadim E. Fraifeld^a^

^a^ The Shraga Segal Department of Microbiology, Immunology and Genetics, Faculty of Health Sciences, Center for Multidisciplinary Research on Aging, Ben-Gurion University of the Negev, Beer-Sheva 8410501, Israel

^b^ Department of Biology of Aging and Experimental Life Span Extension, State Institute of Gerontology of National Academy of Medical Sciences of Ukraine, Kiev 4114, Ukraine

^c^ Departments of Biology and Medicine, Rochester Aging Research Center, University of Rochester, Rochester, NY, 14627, USA

^d^ Department of Molecular Biology, Faculty of Natural Sciences and The Adelson School of Medicine, Ariel University, Ariel 40700, Israel

^#^ These authors contributed equally

* Corresponding author

This article is dedicated in loving memory of Prof. Vadim E. Fraifeld.

**Declaration of interest:** The authors have no competing interests to declare that are relevant to the content of this article

**Funding:** This work was supported by the United States-Israel Research Foundation (BSF;

grant number 2021287 to G.T., V.G., and A.B.). K.K.M. was awarded by Emergency

Fellowships for Ukrainian researchers from The Israel Academy of Sciences and Humanities. E.R. was partially supported by the Israel Ministry of Aliyah and Integration.


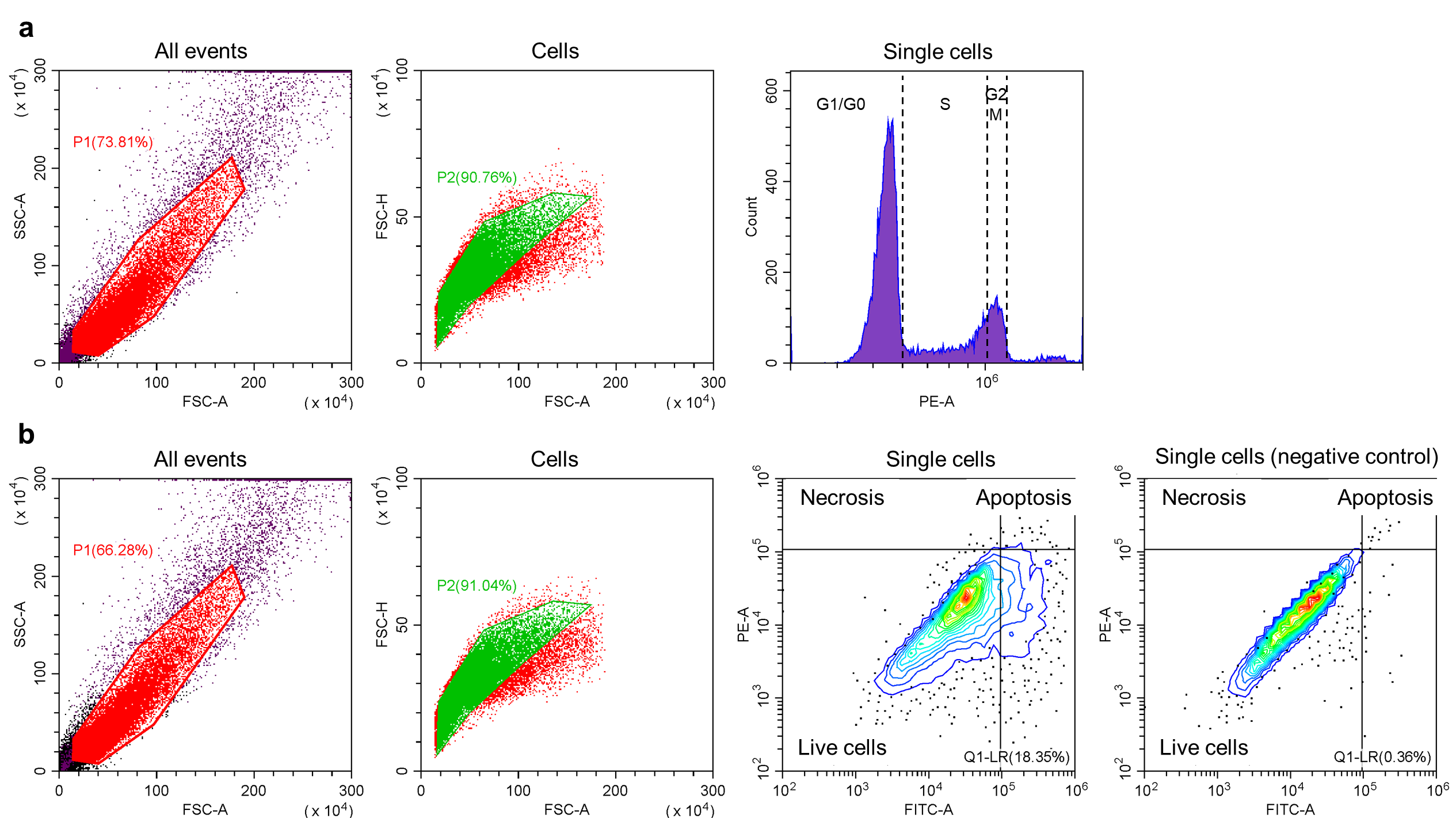


**Figure S1. Gating strategy for FACS analyses.** (**a**) For cell cycle evaluation, the cells were gated based on their size and opacity (FSC-A/SSC-A graph); then the cells were gated based on their shape (only single cells were chosen, FSC-A/FSC-H graph); the gates for phases of cell cycle were selected based on the amount of PI bounded with DNA (PE-A/Count graph). (**b**) For cell death evaluation, the cells were gated based on their size and opacity (FSC-A/SSC-A graph); then the cells were gated based on their shape (only single cells were chosen, FSC-A/FSC-H graph); the gates for necrotic and apoptotic ways of cell death were selected based on the amount of Annexin V on the inner side of cell membrane and PI bounded with DNA (FITC-A/PE-A graph); unstained sample served as negative control.


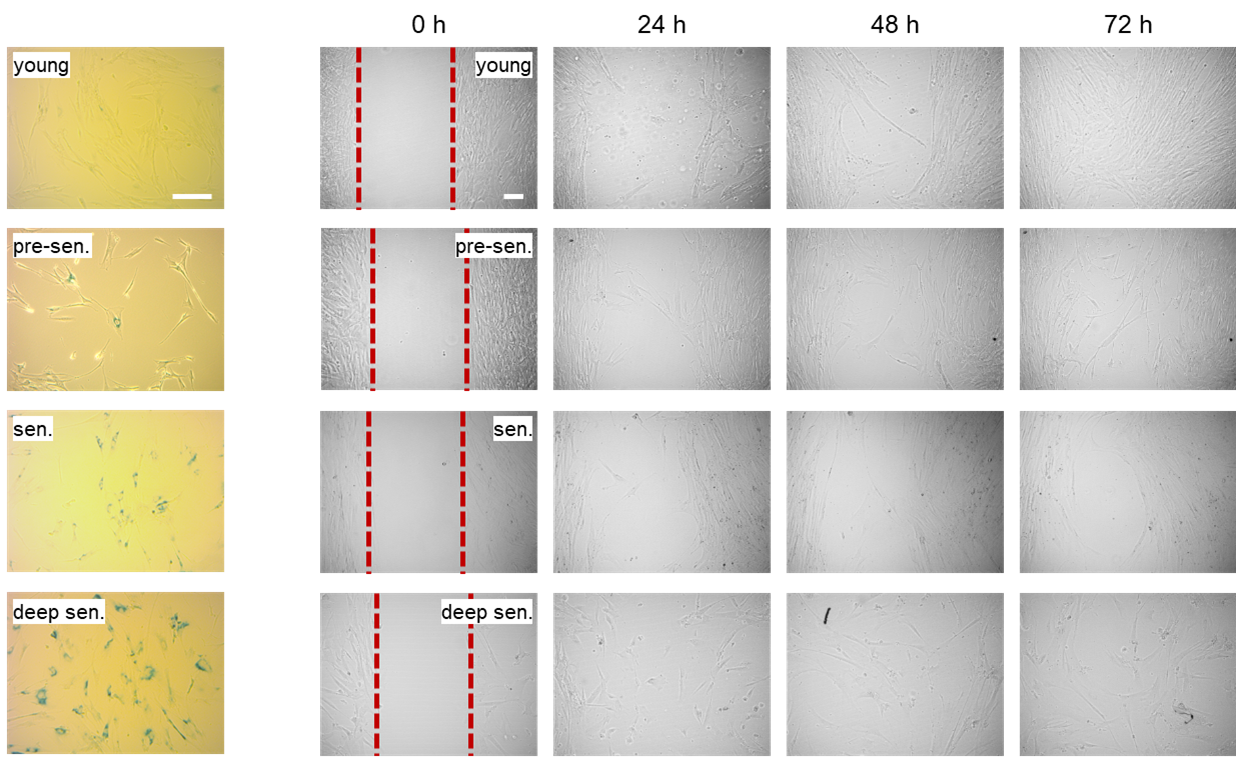


**Figure S2. Representative images of SA-β-gal staining and the gap closure during *in vitro* WH.** Scale bars are 200 μm.
